# Structural effects of 3D printing resolution on the gauge factor of microcrack-based strain gauges for health care monitoring

**DOI:** 10.1038/s41378-021-00347-x

**Published:** 2022-01-27

**Authors:** Sanghun Shin, Byeongjo Ko, Hongyun So

**Affiliations:** 1 Department of Mechanical Engineering, Hanyang University, Seoul, 04763 South Korea; 2 Institute of Nano Science and Technology, Hanyang University, Seoul, 04763 South Korea

**Keywords:** Electrical and electronic engineering, Sensors

## Abstract

Measurements of physiological parameters such as pulse rate, voice, and motion for precise health care monitoring requires highly sensitive sensors. Flexible strain gauges are useful sensors that can be used in human health care devices. In this study, we propose a crack-based strain gauge fabricated by fused deposition modeling (FDM)-based three-dimensional (3D)-printing. The strain gauge combined a 3D-printed thermoplastic polyurethane layer and a platinum layer as the flexible substrate and conductive layer, respectively. Through a layer-by-layer deposition process, self-aligned crack arrays were easily formed along the groove patterns resulting from stress concentration during stretching motions. Strain gauges with a 200-µm printing thickness exhibited the most sensitive performance (~442% increase in gauge factor compared with that of a flat sensor) and the fastest recovery time (~99% decrease in recovery time compared with that of a flat sensor). In addition, 500 cycling tests were conducted to demonstrate the reliability of the sensor. Finally, various applications of the strain gauge as wearable devices used to monitor human health and motion were demonstrated. These results support the facile fabrication of sensitive strain gauges for the development of smart devices by additive manufacturing.

## Introduction

As the importance of smart industrial fields (e.g., human monitoring, the Internet of Things, and soft robotics) has increased in recent times, several state-of-the-art systems have been studied extensively to enhance their efficiency. Strain gauges have been widely used to detect human motion during exercise^1,2^ or talking^3,4^, as well as physiological parameters^5–7^, such as pulse rate, blood pressure, and respiration. Among various strain gauges featuring capacitive monitoring^8–10^, crack-based resistive layers^1,3,5^, and interlocking construction^11,12^, crack-based sensors containing resistive and flexible membranes have attracted significant attention owing to their facile sensing method and simple structure^13,14^.

For the past decade, enormous effort has been devoted to fabricating sensitive crack-based strain sensors, and approaches include determining alternate materials for conductive or flexible layers^1,4,14**–**16^ and varying the structure^16–18^ of flexible sensors. However, combinations of material properties have limitations, and micromachining fabrication entails expensive and complicated processes as well as scalability problems. In addition, to apply current conventional strain sensors to real human monitoring systems, additional packaging procedures integrated with individual applicable hardware, such as gloves or masks^7,19,20^, are required. Therefore, novel and simple methods for fabricating crack-based flexible sensors that simultaneously exhibit high sensitivity for subtle mechanical deformations and potential for scalability are required for the diverse applications of advanced wearable devices.

After the advent of three-dimensional (3D) printing technology, many complicated manufacturing processes were simplified and rendered cost-effective^21–24^. Among various 3D printing methods, the fused deposition modeling (FDM)-based additive manufacturing process is considered the most promising method owing to the range of suitable materials and the rapid fabrication and scalability of the process^22,25**–**27^. However, because of the layer-by-layer stacking process of the FDM method, unwanted rough surfaces are formed that contain groove patterns^28–30^. Although a rough surface is normally thought to degrade printing quality, many studies have utilized regular patterns for other useful applications, such as fabrication of hydrophobic^31^, antiadhesive^32^, and water-harvesting^33^ functional surfaces. Therefore, it is easy to generate specific patterns repeatedly aligned at intervals within the range 50–400 µm by using FDM-based 3D printing. Hence, this approach might replace the conventional microelectromechanical system (MEMS)-based manufacturing processes such as photolithography and etching. In addition, the pattern width can be controlled by changing the thickness of each printing layer, whereas photolithography requires completely new masks or other additional complex methods. Furthermore, mass production without limitations in product shape has opened avenues for various applications.

The gauge factor (GF) of strain sensors, which can be calculated based on resistance changes and mechanical strain, is generally used to represent sensitivity^4,34^. Mechanical deformations caused by external stress open cracks on the metal surface, resulting in an increase in the electrical resistance^19,35^. In particular, to detect subtle signals such as pulse motion, low pressure (<10 Pa), and phonation for human monitoring applications, it is crucial to obtain a high GF value over a small strain range (0–2%) without electrical failure^1,5^. Although high GF values can be obtained using flat strain sensors and additional advanced prestretching processes^3,36,37^, a novel manufacturing method realizing high GFs for strain gauges with complex and customized 3D structures rather than a simple rectangular sheet is required for human monitoring applications.

**Fig. 1:**
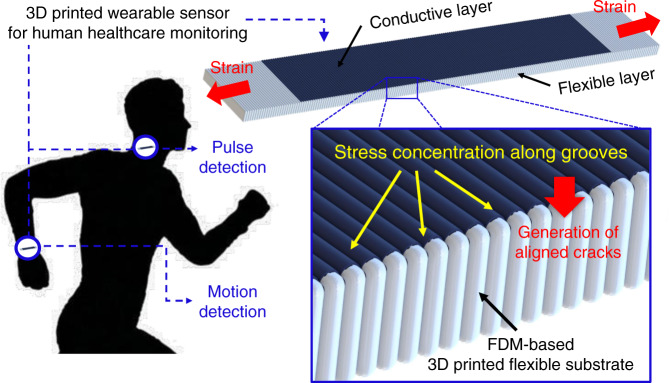
Schematic concept of a 3D printing-based strain gauge containing flexible and conductive layers and its applications. Due to the FDM method, highly aligned crack arrays were easily realized along the groove patterns, resulting in a very sensitive strain gauge

Therefore, in this study, a highly sensitive crack-based strain gauge was fabricated through FDM-based 3D printing and metal sputtering technologies by generating a high density of self-aligned crack arrays with small strain. Figure 1 depicts the schematic concept of the proposed FDM-based strain gauge (FSG). For the flexible layer, thermoplastic polyurethane (TPU), which is known as a biocompatible material^38^, was prepared through 3D printing. In addition, a platinum (Pt) layer was deposited onto the substrate as a conductive layer, and then a prestretching process was used to induce initial crack propagation in advance. Because of the FDM printing method, metal membranes exhibited identical specific groove profiles on their surfaces. Therefore, due to stress concentration, a dense array of cracks was easily formed along the patterns by applying a small strain, which led to a high GF. The fabricated bilayer composite enabled sensitive strain detection through highly aligned cracks. The sensitivity of the flexible FSG was compared for different printing thicknesses, and the characteristics of the cracks generated were also compared. In addition, sensing reliability was demonstrated through structural analysis and transient cycling tests. The 3D printing technology-based manufacturing process is easily applied to prepare various wearable devices that contain strain gauges by simply depositing a metal layer onto the dominant bending area. The findings of this study will facilitate facile fabrication of highly sensitive strain gauges that can be applied to wearable devices for health monitoring and motion detection.

## Fabrication

**Fig. 2:**
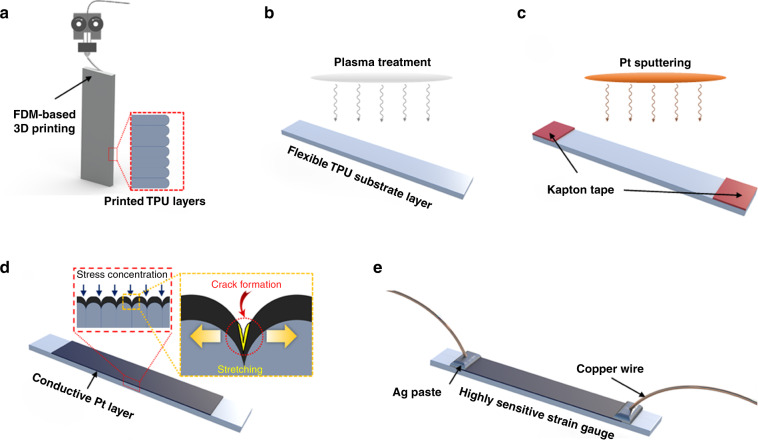
Fabrication process for FDM-based strain gauges. **a** Vertical printing of the TPU substrate for the flexible layer; **b** Preprocessing by plasma treatment before Pt deposition; **c** Pt sputtering onto the exposed TPU layer to produce the conductive layer; **d** Fabricated bilayer composite containing groove patterns; and **e** Electrical connections for resistive sensing

Figure 2 illustrates the overall procedure used for the fabrication of FSGs. As seen in Fig. 2a, a TPU substrate was vertically printed using FDM-based 3D printing (Guider IIs, Flashforge 3D Technology Ltd.) with thickness, width, and height of 1, 6, and 40 mm, respectively. To determine the effects of printing resolution on sensitivity, layer thicknesses of 200, 300, and 400 μm were selected for a 3D printing process. In addition, the printing speed, filling density, bed temperature, and nozzle temperature were set as 20 mm/s, 100%, 60 °C, and 230 °C, respectively. The resulting surfaces of the printed substrates exhibited identical groove patterns for a given printing resolution. Subsequently, air plasma treatment (CUTE-1MPR, Femto Science Inc.) was applied for 30 s to clean the TPU surface before Pt deposition and to ensure better adhesion^5^, as shown in Fig. 2b. Thereafter, a 32-nm-thick Pt layer was deposited onto the TPU surface through an ion sputtering system (E-1045, Hitachi Ltd.), as shown in Fig. 2c. The thickness of the deposited Pt layer was measured by atomic force microscopy (NX20, Park Systems Corp.) installed at Hanyang LINC + Analytical Equipment Center (Seoul). An area (30 mm length and 6 mm width) was exposed for metal deposition through the use of Kapton tape as a sputtering mask. The metal layer on the TPU substrate exhibited identical groove patterns, with stress concentration at the grooves; therefore, cracks can be formed in the deposited metal layer only during stretching, whereas the flexible TPU substrate is not physically damaged (see Fig. 2d). As depicted in Fig. 2e, copper wires and Ag paste were used for electrical connections to evaluate sensing performance in subsequent experiments. Finally, after the entire fabrication, a prestretching process using 2% strain (i.e., 0.6 mm stretching considering the effective 30 mm length of the Pt coating, as shown in Fig. 1) was performed to generate highly aligned crack arrays in advance between each pattern (i.e., valleys of groove patterns).

## Experimental section

To examine the printing quality of the flexible TPU substrate with various printing thicknesses, a digital microscope (UM12, ViTiny, Microlinks Technology Corp.) was used to compare the patterned shapes. Furthermore, to verify the enhanced sensing performance caused by the groove patterns, a TPU substrate without patterns (i.e., flat surface) was also prepared by heating the surface with a heat gun (KX1800, Black & Decker Inc.) and pressing it evenly with a flat glass slide (HSU-1000412, SUPERIOR). In addition, a tensile and compression testing machine (MCT-2150, A&D Co.) was utilized for prestretching and device characterization. The stretching/recovering speed during the test was set as 10 mm/min. Flat-type strain gauges and pattern-type strain gauges with different thicknesses depending on printing resolution (e.g., 200, 300, and 400 μm) were fabricated simultaneously to characterize their sensing performance (i.e., gauge factor and recovery time). To determine the uniformity of the surface profile on the printed TPU substrates, an alpha-step profilometer (Dektak-XT, Bruker) was also utilized. Furthermore, 500 cycling tests were conducted to estimate the reliability of the FSGs. Subsequently, a scanning electron microscope (SEM, S-4800, Hitachi Ltd.) was used to qualitatively compare the formation of cracks for flat- and pattern-type FSGs. Finally, physiological signals from pulse rate, voice, and body motion (i.e., hand and finger motions) were obtained and analyzed to demonstrate the application potential of the fabricated FSGs.

## Results and discussion

**Fig. 3:**
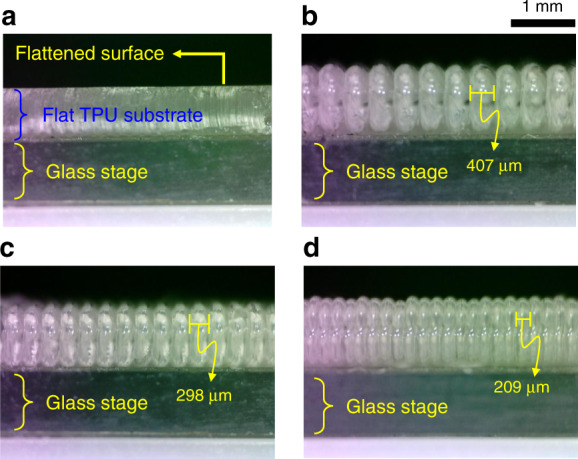
Side-view images of fabricated TPU substrates with various printing resolutions. **a** flat type (without groove patterns); **b** 400 μm; **c** 300 μm; and **d** 200 μm

Figure 3 presents side-view images of four different fabricated flexible substrates placed on the glass stage. The substrates with flat surfaces and layer thicknesses of 400, 300, and 200 μm (hereafter, 4-, 3-, and 2-FSG, respectively) were compared, as shown in Fig. 3a–d, respectively. The compressed flat substrate exhibited an overall smooth top surface. The thicknesses of substrates with TPU layers were 407, 298, and 209 μm for the 4-, 3-, and 2-FSG, respectively. The physical shapes of the Pt surfaces deposited onto the substrates were found to be identical.

**Fig. 4:**
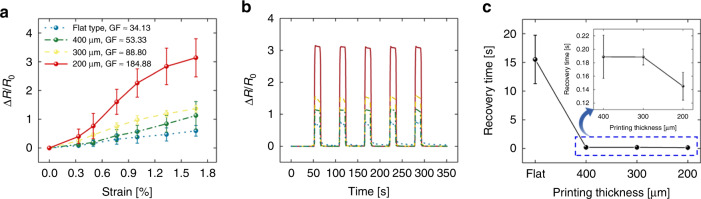
Sensing performance of the fabricated strain gauges. **a** Relative resistance changes depending on various strains; **b** Transient responses with repeated stretching of 0.5 mm; and **c** Recovery time with respect to sensor type

Figure 4 presents the sensing performance results for flat, 4-, 3-, and 2-FSGs. Four different samples were tested for each type of FSG, and their average changes in relative resistance as a function of mechanical stretching length were evaluated and compared. (Fig. 4a). The FSGs were stretched by 0.1, 0.15, 0.23, 0.3, 0.4, and 0.5 mm, which corresponds to mechanical strains of 0.33, 0.5, 0.77, 1, 1.33, and 1.67%, respectively. In addition, average GFs were calculated as 34.13, 53.33, 88.80, and 184.88 for the flat, 4-, 3-, and 2-FSGs, respectively. Because of the high density of crack arrays, the GF of the 2-FSG was ~442% greater than that of the flat type. The results revealed that the smaller the printing layer thickness was, the higher the crack density on the metal surface. Therefore, the rate of change in resistance increased (i.e., enhanced GF). Furthermore, a sensitive strain gauge was easily realized by using a simple fabrication method, and sensitivity could be controlled by tuning the printing thickness during the FDM-based 3D printing process. Figure 4b depicts five repeated transient responses of strain sensors with a stretching range of 0.5 mm. For each sensor, the resistance changed periodically in response to the stretching length and exhibited small standard deviations. In addition, the FSGs with groove patterns exhibited larger resistance change (i.e., were more sensitive) than the flat-type sensor.

For all reversible sensors, the ability to restore the initial signal level rapidly is another important characteristic for continuous sensing applications. To characterize the reversibility of actuation, the recovery time was defined as the time duration between 10% and 90% of the initial value during a 0.5-mm stretching and recovery process. In this experiment, a recovery speed of 300 mm/min was applied to observe instant response results. Figure 4c shows the results for average recovery time with respect to the sensor type. As a result, 2-FSG exhibited a decrease of ~99% compared with that of the flat-type sensor (15.519 and 0.145 s for the flat type and 2-FSG, respectively). It should be noted that all pattern types showed more rapid responses than the flat type. This might be because cracks aligned along the valleys of groove patterns can be rapidly and simultaneously reconnected by the morphological effects of groove patterns, whereas discrete and irregular cracks need some time to close completely. Thus, the 3D-printed substrates exhibited sensitive resistance changes and rapid restoration behavior.

**Fig. 5:**
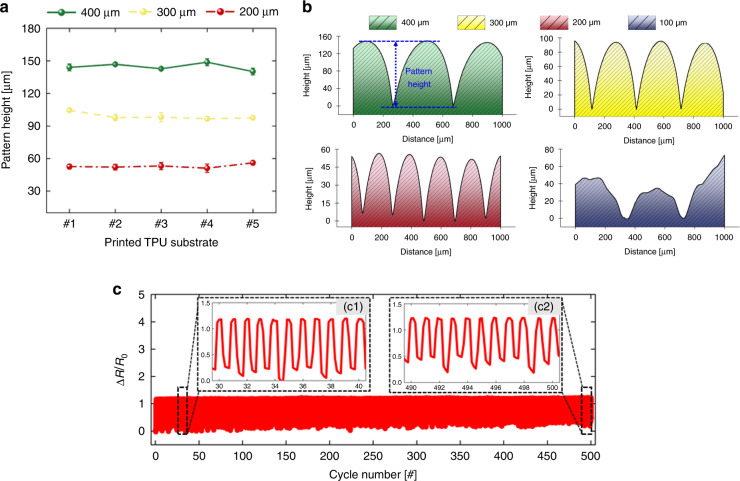
Characterization results of FSGs to demonstrate reliability. **a** Average height of groove patterns for five different printed TPU substrates and **b** their surface profiles with various printing resolutions. **c** Testing of 2-FSG with 500 continuous stretching/recovery cycles and magnified plots. Cycle numbers: (c1) 30 to 40 and (c2) 490 to 500

To demonstrate the uniformity and repeatability of the fabricated strain sensor, surface profile analyses of printed TPU substrates were conducted with different printing resolutions. Figure 5a shows the average for five pattern heights of five different fabricated surfaces depending on printing resolution. As a result, 52.95, 98.93, and 144.52 μm average pattern heights were measured for printing resolutions of 200, 300, and 400 μm, respectively. It was noteworthy that the surface morphology printed by repeated additive manufacturing showed uniform grooves with small standard deviations of 5.7, 5.7, and 3.0% for printing resolutions of 200, 300, and 400 μm, respectively. As the structural characteristics of the 3D-printed TPU surface have a pivotal role in FSGs, this result indicates the reliability of the fabrication method.

Because the GF and recovery time improved as the printing resolution (thickness) decreased, FSGs printed with a resolution below 200 μm showed enhanced GF and recovery time. To investigate the minimum printable resolution, a TPU substrate was printed with 100-μm resolution and compared with other substrates, as shown in Fig. 5b. Compared to the uniform and regular groove patterns seen on substrates printed with 200, 300, and 400-μm resolution, the TPU substrate with 100-μm resolution showed an irregular surface profile. In addition, a comparison of top-view images for the 100-, 200-, 300-, and 400-μm TPU substrates showed incomplete features on the 100-μm TPU substrate (see Figure S1(a) in Supplementary Information), which indicated unstable printing resolution in the FDM printing method used to generate crack-based FSGs. Although the digital light processing (DLP) printing method was used to manufacture a substrate with a smaller printing resolution (25 μm), the substrate was not flexible and showed a relatively smooth surface without groove patterns due to the characteristics of the DLP method, as shown in Figure S1(b); hence, DLP was not appropriate for creating FSGs. Therefore, the minimum printable resolution of 200 μm was selected as the optimal condition for sensitive strain sensors and further analysis, including application examples.

In addition, to evaluate the reversibility and reliability of the 2-FSG, 500 cycling tests were conducted with a uniform stretching length of 0.3 mm in all cycles. As shown in Fig. 5c, the overall sensitivity remained unchanged during the entire test. Furthermore, the fabricated strain gauge repeatedly detected a mechanical deformation of ~1%. The slight deviation in the overall sensitivity, which was also described in previous reports^39,40^, can be minimized by encapsulating the exposed conductive layer^2,13^ (e.g., sandwich structures). These results support the durability and reliability of the FSGs.

**Fig. 6:**
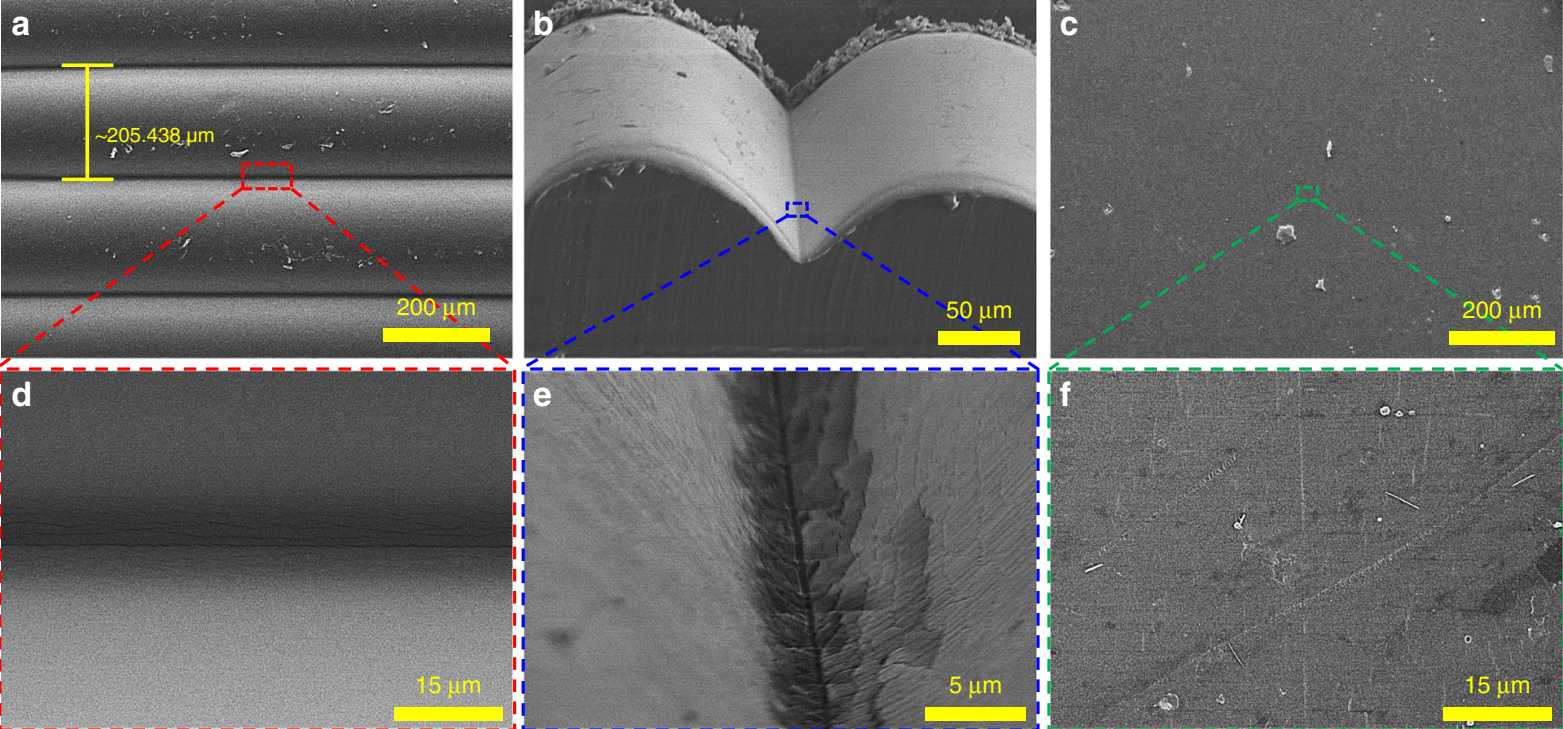
SEM images of prestretched strain gauges to characterize the generated cracks. **a** top and **b** tilted view of 2-FSGs and enlarged views; **d** top and **e** tilted views of a crack along a groove pattern; **c** top view of the flat-type strain gauge and an enlarged view **f**

To qualitatively analyze the structural characteristics of the cracks generated in 2-FSG and the flat strain gauge after prestretching, SEM images of strain gauges in slightly stretched states were captured and compared, as shown in Fig. 6. Figure 6a shows that the crack arrays induced by the groove patterns of FDM-based 3D printing exhibited a high density with an ~205.438 μm gap (pitch distance). Furthermore, highly aligned crack arrays were successfully generated owing to stress concentration along the valleys of the groove patterns, as shown in the magnified view (Fig. 6d). These highly aligned crack arrays improved the GF by allowing efficient disconnection-reconnection of cracks during stretching/recovery processes^16^. In addition, tilted-view SEM images of 2-FSGs were also obtained to analyze the details of crack formation, as shown in Fig. 6b, e. The complete crack formation was achieved along the valley of the groove pattern on the flexible TPU substrate, but the TPU substrate was not physically damaged, as shown in Fig. 6e. Conversely, the flat substrate generated a low density of cracks with ~2% strain (i.e., 0.6 mm stretching length) during prestretching, as shown in Fig. 6c. The crack array in this case was sparse and did not contribute to the high sensitivity of the strain gauge. Furthermore, as observed in Fig. 6e, f, 2-FSG exhibited complete, clear, and straight cracks along the groove pattern, while the flat substrate exhibited incomplete discrete cracks (like a scratch). Therefore, owing to the characteristics of surfaces formed in layer-by-layer deposition of FSGs, even a small strain can generate complete cracks, which contribute to the high sensitivity of the strain gauge^16^.

**Fig. 7:**
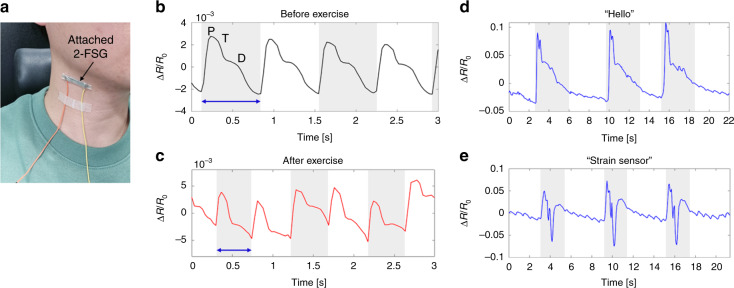
Application for human signal monitoring using 2-FSG. **a** Image of 2-FSG attached to the neck for human monitoring. Pulse rate monitoring **b** before exercise and **c** after exercise. Voice monitoring when repeating **d** “Hello” and **e** “Strain sensor” three times

**Fig. 8:**
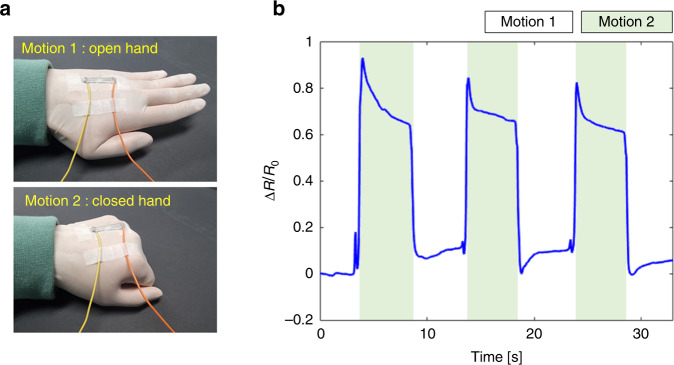
Example of hand motion detection using 2-FSG. **a** Images of an attached 2-FSG for hand motion detection with an open hand (motion 1) and closed hand (motion 2). **b** Detected hand motions repeated three times

Various applications of 2-FSGs have been demonstrated for a range of human monitoring systems. As seen in Fig. 7a, the strain sensor was attached to the neck of a participant (the first author of this manuscript, who provided signed informed consent) to detect their pulse rate and movement of adjacent muscles simultaneously while talking. Figure 7b, c illustrate transient sensing of pulse rates before and after exercise, respectively, without talking. Before exercise, the characteristic wave (P, T, and D waves) was successfully recognized with an average period of ~0.702 s, corresponding to ~85.58 bpm, which lies within the normal range of human pulse rates. Conversely, after exercise, an average period of ~0.474 s (~126.63 bpm) was measured, which was higher than the initial pulse rate (~48% increase). In addition, the pulse pressure (gap between maximum and minimum relative resistance per pulse) also increased by ~62.26% after exercise. Furthermore, two different words (i.e., “hello” and “strain sensor”) were pronounced three times, and the corresponding results are shown in Fig. 7d, e, respectively. Repeated tests with the same word revealed identical muscle movements, for which each signal pattern exhibited the same characteristics. However, different patterns were recorded when a different word was pronounced. In addition, the same pulse patterns were exhibited simultaneously between pronunciations (i.e., during the silent period). To further test the strain gauge for motion detection applications, 2-FSG was attached to the back of the participant’s hand to detect fist-clenching motions. Figure 8 shows the attachment location and the results. For simplification, two types of motion were performed: hand opening (motion 1) and hand closing (motion 2). The transient motion was successfully recognized as a change in relative resistance. The white and green areas in the figure show open (motion 1) and closed hands (motion 2), respectively. When the hand was clenched, a strain was applied to 2-FSG, and the relative resistance increased rapidly. These results confirm the reliable sensing performance of FSG for human health care and motion monitoring applications.

**Fig. 9:**
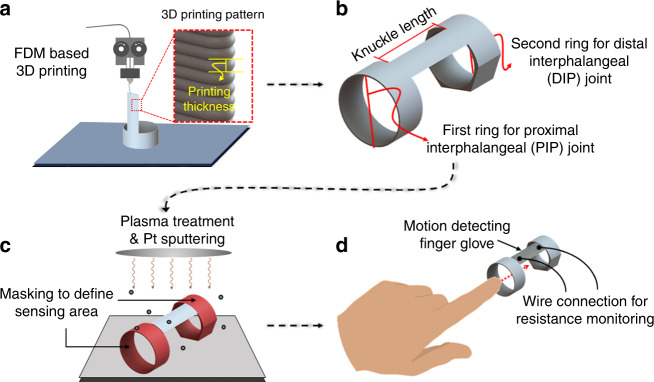
Fabrication process for in situ strain sensing finger gloves. **a** direct 3D printing of a customized finger glove substrate; **b** structural details of the double-ring-shaped band; **c** plasma treatment and Pt sputtering for cleaning and metal deposition processes, respectively; and **d** wire connection and detection of various finger motions by simply wearing the device

**Fig. 10:**
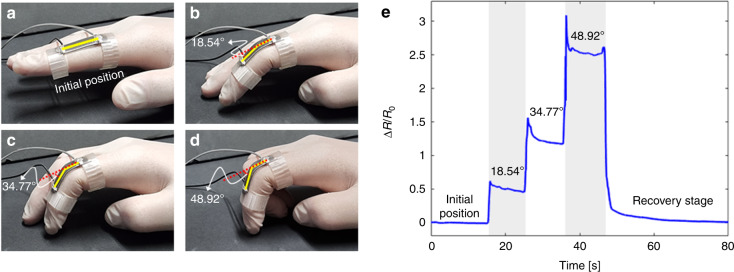
Detection of a single finger motion using an in situ strain sensing finger glove for four different bending motions. **a** initial straight position, **b** 18.54°, **c** 34.77°, and **d** 48.92°. **e** Sensing results for continuous bending/releasing motions

Finally, the 3D printing-based FSG preparation method enabled the facile fabrication of customized wearable devices. To demonstrate a potential application, in situ strain sensing finger gloves were fabricated with selective Pt sputtering at locations of dominant strain. Figure 9 depicts the overall fabrication process for the wearable device. The flexible finger glove made of TPU was directly printed through the FDM method, as shown in Fig. 9a. It is noteworthy that the surface contained distinctive groove patterns that ensured a high GF. In this work, a 200 μm printing thickness was utilized. The geometry of the double-ring-shaped band (Fig. 9b) can be easily modified and customized depending on the physical characteristics of the user (i.e., the radius of distal interphalangeal and proximal interphalangeal joints and length between the joints). Subsequently, the only main stretching part (i.e., the bending area between the rings) was exposed to the plasma treatment and Pt sputtering processes by covering (masking) two rings (Fig. 9c). Finally, the user detected various bending motions of fingers by simply wearing the device with electrical connections. Starting from the initial position with a straight finger (Fig. 10a), the finger was slowly bent to three different angles of 18.54°, 34.77°, and 48.92°, as shown in Fig. 10b–d, respectively. The device detected continuous motions over time, and it recovered to the initial state when the straight finger position was reestablished. These results demonstrated the applicability of the sensor for customized in situ wearable sensing devices (strain gauges) with high sensitivity for various smart systems.

Table 1 shows a comparison of device characteristics, including materials, fabrication method, recovery time, GF, and corresponding strain range, applicable to the present work and previous studies. Compared to chemical synthesis^3,4,13^ and photolithography^16,17^, the formation of self-aligned cracks on a flexible substrate was more easily realized using a 3D printing method without complicated manufacturing processes. In addition, the use of 3D printing technology has the advantage of directly printing customized 3D-shaped substrates, which can be used for finger gloves, wrist bands, and masks where curved surfaces are required, rather than simple rectangular and planar substrates^2,7,15,20^. Although a high GF (~2000) in a small strain range was achieved through advanced prestretching^3^, it was noteworthy that the crack-based strain gauges developed in this study exhibited sufficiently high GFs with faster recovery times compared to other devices^1,5,16^, thus supporting real-time monitoring applications.

**Table 1:** Comparison of various crack-based strain gauges described in the present study and previous studies

Flexible substrate	Conductive layer	Fabrication	Recovery time	GF	Corresponding strain range [%]	Reference
TPU	CNT	Electrospinning	70 ms	428.5	0–100	^1^
TPU	CB	Electrospinning	–	10.8	0–150	^2^
PUA	Pt	Chemical synthesis	–	2000	0–2	^3^
PDMS	CNT	Chemical synthesis	–	87	0–40	^4^
PDMS	Au	E-beam evaporation	> 1 s	200	0–0.5	^5^
PDMS	Ag-rGO	Spray coating	–	7.23	0–0.2	^7^
PDMS	AgNWs	Chemical synthesis	–	30	100	^13^
Ecoflex	Graphite	Laser patterning	–	40	0–30	^15^
PUA	Pt	Lithography	> 1 s	670	0.3	^16^
PDMS	SWCNTs	Lithography	–	161	0–2	^17^
PDMS	AgNWs	Thermal annealing	–	2–14	0–70	^20^
TPU	Pt	3D printing	145 ms	184.88	0–1.67	This work

*PUA Polyurethane acrylate, *PDMS* Polydimethylsiloxane, *CNT* Carbon nanotube, *CB* Carbon black, *Ag-rGO* Graphene oxide decorated with Ag nanoparticles, *SWCNTs* single-wall carbon nanotubes, *AgNWs* Ag nanowires

## Conclusion

Simple 3D printing-based fabrication of highly sensitive strain gauges with small strains (0–1.67%) was demonstrated using FDM-type additive manufacturing with a sputtered metal layer. The printed TPU substrate exhibited a rough surface containing regular groove patterns. The Pt conductive layer subjected to prestretching exhibited highly aligned crack arrays, which contributed to the high sensitivity of the device. The sensing performance was compared for various printing thicknesses, including a flat TPU substrate, to investigate the effects of the groove patterns on GFs and recovery times. The FSG with a thickness of 200 μm (2-FSG) exhibited an ~442% increase in GF and an ~99% decrease in recovery time compared with those of flat-type strain gauges. In addition, the results of surface profile measurements and 500 repeated cycling tests revealed that the strain gauge was reliable. Furthermore, diverse applications for human health care and motion detection were demonstrated successfully. Large (e.g., hand and finger action) and small (e.g., pulse rate and muscle movement) movements with subtle deformations were easily detected. Particularly, by using the facile transformation characteristics of the 3D printing method, an in situ strain sensor (e.g., sensitive finger glove) was fabricated for human motion detection. The findings of this study will facilitate facile additive manufacturing of highly sensitive 3D printing-assisted strain sensors for human monitoring systems.

## Supplementary information

Supplemental Material
