# Supplementary material for: Structural effects of 3D printing resolution on the gauge factor of microcrack-based strain gauges for health care monitoring
Source: Microsyst Nanoeng. 2022 Jan 27;8:12. doi: 10.1038/s41378-021-00347-x (PMC8791987; doi:10.1038/s41378-021-00347-x)
Supplement: Supplementary file 1 — Supplemental Material [file 41378_2021_347_MOESM1_ESM.docx]

**Structural effect of 3D printing resolutions on gauge factor of microcrack-based strain gauges for healthcare monitoring**

Sanghun Shin^1^, Byeongjo Ko^1^, and Hongyun So^1,2,^[[1]](#footnote-1)^a)^

^1^Department of Mechanical Engineering, Hanyang University, Seoul 04763, South Korea

^2^Institute of Nano Science and Technology, Hanyang University, Seoul 04763, South Korea

***Keywords:*** *strain gauges, flexible sensors, 3D printing, wearable device, healthcare*

**S1. Surface characteristic comparison**

**S2. Adhesion test**

**S1. Surface characteristic comparison**

Comparison of fabricated substrates with different printing conditions (printing resolutions and printing type) was conducted in this study. For FDM printing method, a printing resolution of 100, 200, 300, and 400 μm was used. For digital light processing (DLP) printing method (Hunter, Flashforge 3D Technology Ltd.), a printing resolution of 25 μm with UV curable resin (FH1100, Flashforge Standard Resin) was employed to create the FSG substrate. Figure S1(a) demonstrates top-view optical images of printed surfaces using FDM and DLP printing methods. To demonstrate the presence of regular groove patterns on the same sized substrate, the rectangular substrates (6 mm × 40 mm) were printed and compared. As a result, the TPU substrate with 100-μm resolution showed incomplete surface feature and groove patterns. In addition, the substrate printed using DLP method showed relatively smooth profile without groove patterns, as shown in Fig. S1(b), indicating that the FDM printing is more suitable for manufacturing of crack-based FSGs.





**Figure S1.** Surface characteristic depending on printing resolution and method. (a) Optical top view of FDM-based printed TPU substrates and DLP-based printed UV curable resin. (b) Side view of DLP-based printed substrate and the surface profile measured by surface profiler.

**S2. Adhesion test**

As the adhesive force between the metal (Pt) layer and polymer (TPU) substrate is crucial for durability, peeling-off adhesion tests were conducted. For the experiments, 3M scotch tape was used. Figure S2(a) shows a schematic of the test process. After the tape was attached on top of the 2-FSG surface, the tape was pulled at peeling angles (θ) of 0° and 90° with 300 mm/min speed using a tester. If the conductive metal layer is physically damaged due to the weak adhesion force between Pt and TPU surface, the electrical resistance will significantly increase. Therefore, the changing rates of relative resistance compared to the initial value was measured at each test iteration. As shown in Fig. S2(b), for the case of θ=0°, the changing rate of relative resistance hardly changed (only ~1.23% increased after five tests). Although the relative resistance changing rate in case of θ=90° increased by ~3.94% after five tests due to the relatively stronger peeling-off force depending on the peeling angle [1], the change was very little, indicating a good adhesion between Pt and TPU. Furthermore, optical images of the metal surface after the tests (see inset images in Fig. S2(b)) showed no noticeable and significant physical defects, demonstrating the durability of the FSGs.

|  |
| --- |
| **Figure S2.** Pt/TPU adhesion test using 2-FSG. (a) Schematic of peeling test using 3M tape and 2-FSG. (b) Relative resistance changing rate during five repeated tests with different two peeling angles (0º and 90º). |

[1] Z. Peng, C. Wang, L. Chen, S. Chen, Peeling behavior of a viscoelastic thin-film on a rigid substrate, Int. J. Solids Struct. 51 (2014) 4596–4603.

1. a) Author to whom correspondence should be addressed. E-mail: hyso@hanyang.ac.kr [↑](#footnote-ref-1)
